# LncRNA AGAP2 antisense RNA 1 stabilized by insulin-like growth factor 2 mRNA binding protein 3 promotes macrophage M2 polarization in clear cell renal cell carcinoma through regulation of the microRNA-9-5p/THBS2/PI3K-Akt pathway

**DOI:** 10.1186/s12935-023-03173-5

**Published:** 2023-12-18

**Authors:** Peng Xu, Da-xiong Feng, Jun Wang, Yao-Dong Wang, Gang Xie, Bin Zhang, Xiao-Han Li, Jia-Wei Zeng, Jia-Fu Feng

**Affiliations:** 1 NHC Key Laboratory of Nuclear Technology Medical Transformation (MIANYANG CENTRAL HOSPITAL), Mianyang Central Hospital, School of Medicine, University of Electronic Science and Technology of China, No. 12 Changjia Lane, Jingzhong Street, Mianyang, Sichuan 621000 People’s Republic of China; 2 Department of Clinical Laboratory, Mianyang Central Hospital, School of Medicine, University of Electronic Science and Technology of China, Mianyang, 621000 People’s Republic of China; 3 Department of Spine Surgery, The Affiliated Hospital of Southwest Medical University, Luzhou, 646000 People’s Republic of China; 4 Department of Laboratory Medicine, Sichuan Provincial Maternity and Child Health Care Hospital, Chengdu, 610045 People’s Republic of China; 5 Department of Urology Surgery, Mianyang Central Hospital, School of Medicine, University of Electronic Science and Technology of China, Mianyang, 621000 People’s Republic of China; 6 Department of Pathology, Mianyang Central Hospital, School of Medicine, University of Electronic Science and Technology of China, Mianyang, 621000 People’s Republic of China; 7 Department of Medical Laboratory, The Affiliated Hospital of Southwest Medical University, Luzhou, 646000 People’s Republic of China

**Keywords:** Renal cell carcinoma, N6-methyladenosine, Long non-coding RNA, AGAP2-AS1, IGF2BP3, miR-9-5p, THBS2, PI3K/AKT, M2 polarization

## Abstract

**Background:**

Increasing evidence highlights the potential role of long non-coding RNAs (lncRNAs) in the biological behaviors of renal cell carcinoma (RCC). Here, we explored the mechanism of AGAP2-AS1 in the occurrence and development of clear cell RCC (ccRCC) involving IGF2BP3/miR-9-5p/THBS2.

**Methods:**

The expressions of AGAP2-AS1, IGF2BP3, miR-9-5p, and THBS2 and their relationship were analyzed by bioinformatics. The targeting relationship between AGAP2-AS1 and miR-9-5p and between miR-9-5p and THBS2 was evaluated with their effect on cell biological behaviors and macrophage polarization assayed. Finally, we tested the effect of AGAP2-AS1 on ccRCC tumor formation in xenograft tumors.

**Results:**

IGF2BP3 could stabilize AGAP2-AS1 through m6A modification. AGAP2-AS1 was highly expressed in ccRCC tissues and cells. The lentivirus-mediated intervention of AGAP2-AS1 induced malignant behaviors of ccRCC cells and led to M2 polarization of macrophages. In addition, THBS2 promoted M2 polarization of macrophages by activating the PI3K/AKT signaling pathway. AGAP2-AS1 could directly bind with miR-9-5p and promote the expression of THBS2 downstream of miR-9-5p. These results were further verified by in vivo experiments.

**Conclusion:**

AGAP2-AS1 stabilized by IGF2BP3 competitively binds to miR-9-5p to up-regulate THBS2, activating the PI3K/AKT signaling pathway and inducing macrophage M2 polarization, thus facilitating the development of RCC.

**Supplementary Information:**

The online version contains supplementary material available at 10.1186/s12935-023-03173-5.

## Introduction

AGAP2-AS1 is an antisense lncRNA transcribed from the 12q14.1 gene, with a length of 1567 nt [1]. Studies have shown that AGAP2-AS1 is associated with the development of various cancers, including gastric cancer [2], non-small cell lung cancer (NSCLC) [3], breast cancer [4], pleomorphic glioblastoma [5], ovarian cancer [6], pancreatic cancer [7], and hepatocellular carcinoma (HCC) [8]. Increasing evidence highlights the potential role of long non-coding RNAs (lncRNAs) in the biological behaviors of renal cell carcinoma (RCC). Statistics indicate that RCC affects more than 400,000 persons every year worldwide [9]. Clear cell RCC (ccRCC) represents about 80% of RCC [9]. Although early ccRCC can be treated by surgery or ablation, over one-third of cases have recurrence or distant metastasis. In addition, its lack of sensitivity to chemotherapy and radiotherapy has prompted studies to continuously explore the molecular mechanism of ccRCC and new therapeutic targets/strategies [10].

Non-coding RNA (lncRNA) plays a vital role in epigenetic regulation and can regulate gene expression [11]. LncRNA AGAP2 antisense RNA 1 (AGAP2-AS1) is a newly identified cancer-related antisense lncRNA located at 12q14. 1. It is dysregulated in various tumor cells (such as lung, liver, gastric, breast, and pancreatic tumors) and is closely related to the prognosis of tumor patients [12–14]. Previous studies have indicated that AGAP2-AS1, stimulated by E2F4, aggravates the development of colorectal cancer (CRC) by regulating the miR-182-5p/CFL1 axis. The high expression of AGAP2-AS1 in CRC suggests its potential as an effective new target for future CRC treatment [15]. Additionally, AGAP2-AS1 exhibits high expression in gliomas, where it may function as an oncogene by inhibiting the expression of miR-497-5p, thus highlighting its potential as a prognostic biomarker and therapeutic target in gliomas [16]. As previously documented, AGAP2-AS1 may be a clinical biomarker for subsequent tumor progression in "low-risk" ccRCC patients [17], and it may also serve as an independent predictor of unfavorable outcomes of ccRCC patients [18]. However, the functional significance of AGAP2-AS1 in the pathophysiology of ccRCC remains undefined.

N6-methyladenosine (m6A) is the most common post-transcriptional modification in eukaryotic RNA, accounting for about 80% of RNA methylation modifications [19, 20], and related to the occurrence of many diseases, especially cancer [21, 22]. The m6A RNA methylation bears excellent responsibility for ccRCC progression, and m6A-related lncRNAs can accurately predict the prognosis of patients with ccRCC [23, 24]. In addition, m6A regulators may be used for the prognostic prediction of ccRCC [25].

Insulin-like growth factor 2 mRNA binding proteins (IGF2BPs) are members of the conserved RNA-binding oncofetal proteins, consisting of three members: IGF2BP1, IGF2BP2, and IGF2BP3 [26]. IGF2BP3 is closely related to the invasion, malignancy degree and prognosis of human tumors [27–29]. More importantly, IGF2BP3 regulates the transcription of NUF2 by stabilizing CDKN2B-AS1, thereby enhancing the malignancy of ccRCC [30]. Recent studies have found that IGF2BP3 overexpression aggravates RCC [30–32].

MicroRNAs (miR) are a group of short, single-stranded, and non-coding RNAs involved in regulating gene expression [33]. MiR-9-5p, a processing product of 5 prime of miR family member miR-9, also plays a role in promoting cancer or inhibiting cancer in many tumors, including cervical cancer, liver cancer [34], prostate cancer [35], breast cancer [36] and RCC [37] etc. Thrombospondin 2 (THBS2), a member of the calcium-binding glycoprotein family of stromal cells, is crucially involved in cell proliferation, adhesion and apoptosis. Many studies have found that THBS2 is highly expressed in different cancers, including RCC [38–40]. The phosphatidylinositol-3 kinase (PI3K)/protein kinase B (Akt) pathway plays an important role not only in tumor progression but also in tumor response to cancer treatment [41–43]. Many studies have shown that the proteins involved in PI3K/Akt signal transduction are prone to high expression in patients with RCC, promoting tumor progression and metastasis [42, 44, 45]. Despite these studies, the exact role of miR-9-5p/THBS2/PI3K-Akt in RCC remains unclear.

M2 macrophage is a type of tumor-associated macrophage, also known as alternately activated macrophages, which promotes the occurrence and development of tumors [46], including RCC [47, 48]. A recent study has highlighted the role of the Akt pathway in the polarization of macrophages, thereby affecting tumor progression [49]. Therefore, we speculate that miR-9-5p/THBS2/PI3K-Akt axis is a potential mechanism of M2 macrophage polarization in RCC. In summary, this study has revealed the role of IGF2BP3 in stabilizing AGAP2-AS1 expression through m6A modification. This establishment of expression then triggers M2 polarization of macrophages via the miR-9-5p/THBS2/PI3K-Akt signaling pathway, ultimately promoting the onset and progression of RCC. These findings provide a novel theoretical basis for the study of RCC and offer potential therapeutic targets, early diagnostic markers, and immunotherapy strategies. Such discoveries hold significant implications for improving patient prognosis and treatment outcomes. However, further research and clinical validation are still necessary to ensure the practical impact of these findings on patient care.

While this study reveals important insights, it has limitations. It primarily relies on in vitro experiments and mouse models, necessitating validation in humans. Understanding IGF2BP3's role in AGAP2-AS1 regulation and macrophage polarization in RCC remains incomplete. Variations in these molecules across RCC subtypes and their impact on disease progression warrant further investigation. Additionally, AGAP2-AS1's multifaceted roles in various cancers require deeper exploration. The specific molecular mechanisms and their roles in different malignancies remain unclear. Future research should further explore AGAP2-AS1's multifunctionality and regulatory mechanisms across diverse cancer types.

Furthermore, while this study establishes a foundation for early RCC diagnosis and treatment, translating these findings into practical clinical applications requires additional effort. The critical step involves conducting clinical trials and patient cohort studies to confirm the feasibility and effectiveness of these findings in a clinical context. Ultimately, these endeavors hold the potential to significantly benefit the treatment and management of RCC patients.

## Materials and methods

### Ethics approval and consent to participate

All experiments were conducted according to the ethical guidelines of the Ethics Committee of Mianyang Central Hospital and strictly followed the "Declaration of Helsinki". All patients signed written informed consent. The Animal Ethics Committee of Mianyang Central Hospital has approved the experimental procedure and animal use protocol.

### Bioinformatics analysis

The GSE36895 and GSE40435 datasets were from the GEO database. The GSE36895 dataset contains 23 standard kidney tissue samples and 29 ccRCC samples, while the GSE40435 dataset contains 101 ccRCC samples and 101 standard tissue samples. The differentially expressed genes were screened using the R language "limma" package with |logFC|> 1 and P value < 0.05 as the screen criteria. The expression of target genes in ccRCC was analyzed in the ENCORI database. The m6A2Target was used to predict the target lncRNAs of m6A. The LncBase database was used to predict the miRNAs targeted by lncRNAs. The TargetScan database was applied for the prediction of miRNA target genes. The KOBAS database was used for KEGG analysis.

### Sample collection

The 50 pairs of ccRCC and para-cancerous tissues were collected from 50 ccRCC patients who were surgically treated in Mianyang Central Hospital from March 2020 to May 2021. None of the patients had received radiotherapy before specimen collection. The tumor T-stage classification was based on the 2010 TNM classification, and the tumor grading was based on the Fuhrman classification. The clinicopathological characteristics of patients are shown in Additional file 3: Table S1.

### Reverse transcription-quantitative polymerase chain reaction (RT-qPCR)

Issues and cellular total RNA were extracted using Trizol (Catalog No. 16096020, Thermo Fisher Scientific, New York, USA). mRNA was reverse transcribed to cDNA using a reverse transcription kit (Catalog No. RR047A, Takara, Japan). miRNA was synthesized to cDNA using a PolyA tailing kit (Catalog No. B532451, Shenggong, China). The reaction system was prepared using the SYBR Premix Ex TaqTM II kit (Catalog No. DRR081, Takara, Japan), and real-time quantitative PCR (RT-qPCR) was performed on a real-time fluorescence quantitative PCR instrument (ABI 7500, ABI, Foster City, CA, USA) with the following program: 10 min at 95 °C, followed by 35 cycles of 15 s at 95 °C, 30 s at 60 °C, and 45 s at 72 °C. U6 was used as the reference gene for miRNA, and GAPDH was used as the reference gene for other genes. All RT-qPCR reactions were performed in triplicate, and the experiment was repeated three times. The relative gene expression was calculated with the 2^−ΔΔCt^ method [50]. The primers are listed in Additional file 3: Table S2.

### Cell lines and cell culture

Human renal tubular epithelial cell line HK-2, human RCC cell lines 786-O and ACHN, human embryonic kidney cell line 293 T, and human leukemia monocyte cell line THP-1 were obtained from ATCC (USA). HK-2 cells were cultured in DMEM/F12 (A4192001, Gibco, USA) medium, 786-O and THP-1 cells were cultured in RPMI1640 (22400089, Invitrogen, USA) medium, and ACHN and 293 T cells were cultured in DMEM (10569044, Gibco, USA) medium. All culture media were supplemented with 10% FBS (100099141, Gibco, USA) and 1% penicillin–streptomycin (15070063, Gibco, USA). Cells were cultured at 37 °C with 5% CO_2_ in a cell culture incubator and passaged at a 1:3 ratio using 0.25% trypsin for cell dissociation. The PI3K/AKT inhibitor LY294002 (HY-10108, MedChemExpress) was dissolved in DMSO and used at a final concentration of 10 μM to treat ACHN and 786-O cells for 2 h. The methylation inhibitor 3-deoxyadenosine (HY-W013332, MedChemExpress, USA) was prepared in DMSO to obtain a final concentration of 50 mM and applied to cells for 24 h. THP-1 cells (1 × 10^6^ cells) were induced to differentiate into macrophages by incubating with 100 ng/mL PMA (Sigma-Aldrich, USA) for 24–48 h. After washing the macrophages with PBS, they were co-cultured with ccRCC cells for 2 days.

### Cell transduction

The cells in the logarithmic growth phase were seeded in a 6-well plate at a density of 4 × 10^5^ cells per well. When the cells reached 70–80% confluence, the culture medium was replaced with a serum-free medium. Transfection was performed using lipo2000 (11668-019, Invitrogen, USA). Specifically, 4 μg DNA was dissolved in 250 μl Opti-mem serum-free medium in tube A, while 10 μl lipo2000 was dissolved in 250 μl Opti-mem serum-free medium in tube B and left at room temperature for 5 min. The tubes A and B contents were gently mixed and incubated for 20 min before adding to the 6-well plate. The plasmid transfection groups included the Vector group (transfected with pcDNA3.1 empty vector), IGF2BP3 group (overexpressing IGFBP3, transfected with pcDNA3.1-IGFBP3 plasmid), IGF2BP3-KH3/4-Mut group (overexpressing IGFBP3 with KH3/4 domain mutation, transfected with pcDNA3.1-IGFBP3-KH3/4-Mut plasmid), and AGAP2-AS1 group (overexpressing AGAP2-AS1, transfected with pcDNA3.1-AGAP2-AS1 plasmid). Each plasmid was transfected at a dose of 4 μg, and both the transfection sequences and plasmids were purchased from Shanghai Gene Biotechnology Co., Ltd.

### Lentiviral infection

The lentivirus transduction and grouping were conducted as follows: Lentiviruses for gene silencing were packaged using the core plasmid (pLKO.1) containing the silencing sequence and the auxiliary plasmids (psPAX2, pMD2.G). Lentiviruses for gene overexpression were packaged using the core plasmid (pHAGE-CMV-MCS-IzsGreen) containing the cDNA sequence of the target gene and the auxiliary plasmids (psPAX2, pMD2.G). Lentiviruses were purchased from Genomeditech in Shanghai. The packaged viruses and the target vectors were co-transfected into 293 T cells using Lipo2000. After 48 h of cell culture, the supernatant was collected, filtered, and centrifuged to obtain virus particles. The viral titer was measured. Viruses harvested during the logarithmic growth phase were divided into the following groups: sh-NC (silencing lentivirus control group), sh-AGAP2-AS1-1 (silencing AGAP2-AS1 group 1), sh-AGAP2-AS1-2 (silencing AGAP2-AS1 group 2), sh-THBS2-1 (silencing THBS2 group 1), sh-THBS2-2 (silencing THBS2 group 2), oe-NC (overexpression lentivirus control group), oe-THBS2 (overexpression THBS2 group), mimic NC (miR-9-5p overexpression control group), miR-9-5p mimic (miR-9-5p overexpression group), inhibitor NC (miR-9-5p inhibitor control group), and miR-9-5p inhibitor (miR-9-5p inhibitor group). Vector (empty lentivirus-infected group), IGF2BP3-WT (lentivirus-infected group overexpressing cDNA sequence of IGF2BP3), IGF2BP3-KH3/4-Mut (lentivirus-infected group with a point mutation introduced in the cDNA fragment of IGF2BP3's KH3-4 domain, packaged as an overexpression vector), IGF2BP3-KH3/4-Mut + DAA (lentivirus-infected group with IGF2BP3-KH3/4-Mut and treatment with the methylation inhibitor 3-deoxyadenosine (DAA) at a concentration of 50 µm for 24 h. When cells reached the logarithmic growth phase, they were dissociated using trypsin, resuspended to a concentration of 5 × 10^4^ cells/mL, and seeded in a 6-well plate with each well containing 2 mL of the cell suspension. The plate was then incubated overnight at 37 °C. After 48 h of infection, the expression levels of the target genes in each group were determined by RT-qPCR, with three replicates.

### RNA binding protein immunoprecipitation (RIP)

AGAP2-AS1 was pulled down using an IGF2BP3 antibody (ab177942, 1:50, Abcam). Subsequently, the IGF2BP3 antibody was recovered using protein A/G beads (Santa Cruz, USA). The RNA levels of AGAP2-AS1 in the sediment were determined by RT-qPCR analysis. The m6A levels of AGAP2-AS1 were assessed using the MeRIP m6A kit (Merck Millipore, Schwarbach, Germany). Methylation-specific RNA immunoprecipitation (MeRIP) analysis was performed following the instructions provided with the kit. Subsequently, RNA extraction and RT-qPCR analysis were performed to evaluate AGAP2-AS1 expression [46].

### RNA pull-down assay

The WT-miR-9-5p (wild-type) and MUT-miR-9-5p (mutant-type) were synthesized and purified in vitro. Biotin RNA Labeling Mix (Roche, Switzerland) and T7 RNA polymerase (Ambion Life) were utilized to label the synthesized miRNAs with biotin. The labeled RNA samples were recovered using the RNeasy Plus Mini Kit (Qiagen, Valencia, CA, USA) and DNase I (Qiagen). The purified RNA samples were incubated with cell lysates. Subsequently, magnetic beads were added to the samples and incubated at room temperature. The RNA complexes bound to the magnetic beads were then eluted and subjected to q-PCR analysis [51].

### RNA half-life detection

The cells were treated with Actinomycin D (129935, Millipore) at 5 μg/ml. After 0, 4, 8 and 12 h of incubation, the cells were harvested, and RNA was extracted for PCR detection. The RNA degradation rate was calculated: NtN0 = e^−kt^ (where t is the transcription inhibition time, and Nt and N0 are the RNA expression levels at time t and time 0). RNA lifetime (t_1/2_) was calculated from the following degradation rate: t_1/2_ = ln_2_k [52].

### Dual-luciferase reporter assay

Firstly, we obtained potential binding sites between AGAP2-AS1 and miR-9-5p, as well as between miR-9-5p and THBS2, from the TargetScan and StarBase databases. We inserted cDNA fragments containing the binding sites between AGAP2-AS1 and miR-9-5p and THBS2 and miR-9-5p into the pmirGLO vector. The AGAP2-AS1-MUT and THBS2-MUT cDNA fragments, carrying point mutations, were also synthesized and inserted into the pmirGLO vector. We constructed pmirGLO-AGAP2-AS1-WT (5'-AATATTTCTTAAACTACCAAAGG-3') and pmirGLO-AGAP2-AS1-MUT (5'-ATATTTTGATAAACTAGGTTTCG-3'), as well as pmirGLO-THBS2-WT (5'-ACGTCATGTGTTTTGCCAAAGAC-3') and pmirGLO-THBS2-MUT (5'-ACGTCATGTGTTTTGCGGTTTCC-3') plasmids. The plasmids above and miR-9-5p mimic and mimic NC plasmids were transfected into 293 T cells using lipofection. After 48 h of transfection, the cells were lysed, and the supernatant was collected by centrifugation at 12,000 rpm for 1 min. The Firefly luciferase activity was measured using the luminometer assay kit (K801-200, Biovision, USA), followed by detection of the luminescence using the Dual-Luciferase Reporter Assay System (E1910, Promega). For each cell sample, 100 μL of Firefly luciferase working solution was added to measure the Firefly luciferase (FI/Rely luciferase), and 100 μL of Renilla luciferase working solution was added to measure the Renilla luciferase. luc2 represents the intensity of the Firefly luciferase reaction, while hRluc-neo represents the intensity of the internal control Renilla luciferase reaction. The ratio of luc2/hRluc-neo was calculated from the obtained data. Each experiment was performed in triplicate [48].

### Cell viability by CCK-8

The CCK-8 kit (K1018, Apexbio, USA) was used to evaluate the cell viability after lentivirus infection. Cells (1.0 × 10^4^ per well) were plated in a 96-well plate (100 μL/well). After culturing for 12 h, 24 h, 36 h, and 48 h, 10μL of CCK-8 solution was added. The absorbance at 450 nm was measured with a microplate reader [47].

### EdU cell proliferation assay

The cells were seeded in a 24-well plate and added with EdU (ST067, Beyotime, Shanghai, China) at 10 µmol/L concentration. After incubation for 2 h, cells (6–10 fields) were randomly observed under a fluorescence microscope (FM-600, Shanghai Pudan Optical Instrument Co., Ltd.). The number of EdU-positive cells in each field was counted [53]. EdU labeling rate (%) was calculated as the number of positive cells/(number of positive cells + number of harmful cells) × 100%.

### Transwell invasion assay

The Transwell chamber (Corning, USA) with 8 μm pore size was used to conduct an in vitro cell invasion assay in a 24-well plate. The upper chamber of the Transwell chamber was coated with diluted Matrigel (diluted 1:8 in serum-free medium) and incubated at 37 °C for 1–4 h to allow Matrigel gelation. In the lower chamber of the Transwell chamber, 600 mL of 20% FBS-containing medium was added and equilibrated at 37 °C for 1 h. Cells treated differently were resuspended in a serum-free medium and seeded at a concentration of 1 × 106/mL in the upper chamber. The cells were then incubated at 37 °C and 5% CO_2_ for 24 h. After incubation, the Transwell chamber was removed and washed twice with PBS. The cells were fixed with 5% glutaraldehyde at 4 °C, stained with 0.1% crystal violet for 5 min, rinsed twice with PBS, and surface cells were wiped off with a cotton pad. The cells were observed under an inverted fluorescence microscope (Nikon, China, model TE2000). Five random fields were selected and photographed for each group, and the average number of cells that had invaded the chamber was determined. Each experiment was repeated three times [36].

### Wound healing assay

Lines were evenly drawn every 0.5–1 cm on the bottom surface of a 6-well plate using a ruler and marker pen, with at least 5 lines passing through each well. 5 × 10^5^ cells/well were added to the six-well plate and cultured overnight in a complete medium. The next day, a scratch was made perpendicular to the back lines, using a 200 μl pipette tip, and the medium was replaced with serum-free medium. The distances of the scratch were measured and recorded under an optical microscope (Leica, model DM500) at 0 h and 24 h. Images were captured using an inverted microscope to observe the migration ability of the cells in each group. The wound healing rate was calculated as (T0 area—Tt area) / T0 area × 100% [47].

### *Fluorescence *in situ* hybridization (FISH)*

The AGAP2-ASA and miR-9-5p localization in cells was determined using the FISH technique. RiboTM lncRNA FISH Probe Mix (Red) (Ruibo Biotechnology, China) was used according to the manufacturer's instructions [47]. The specific method was as follows: coverslips were placed in a 6-well culture plate, and the test cells (1 × 105 cells/well) were seeded into the wells and incubated for 1 day to reach a cell fusion rate of around 80%. The coverslips were removed, washed with PBS, and fixed with 1 mL of 4% paraformaldehyde at room temperature. After treatment with proteinase K (2 μg/mL), glycine, and acetylation reagent, 250 μL of pre-hybridization solution was added and incubated at 42 °C for 1 h. The pre-hybridization solution was removed, and 250 μL of hybridization solution containing the probes (300 ng/mL) was added and hybridized overnight at 42 °C. After washing three times with PBST, the nuclei were stained with DAPI diluted in PBST (1:800) and incubated for 5 min in a 24-well culture plate. The samples were then washed three times with PBST for 3 min each. Finally, the coverslips were mounted with an anti-fluorescence quenching agent and observed and photographed using a laser confocal microscope (Leica, TCS-SP8 SR, Germany) with five fields of view selected for analysis.

### Western blot

The total protein extracts were separated by electrophoresis and transferred to a polyvinylidene fluoride membrane (1620177, BIO-RAD, USA). After that, the membrane was incubated with primary antibodies, including mouse anti-β-actin (#8226, 1:5000, CST, US), rabbit anti-THBS2 (ab112543, 1:1000, Abcam, UK), rabbit anti-PI3K (#4292, 1: 1000, Cell Signaling Technology), rabbit anti-p-PI3K (phospho Y607) (ab182651, 1:800, Abcam), rabbit anti-ATK (#9272, 1:1000, CST, United States), and rabbit anti-p-AKT (phospho S473) (#9271, 1:1000, CST, USA). After washing, the secondary antibody of HRP-labeled goat anti-rabbit IgG (ab6721, 1:5000, Abcam) or goat anti-mouse IgG (ab205719, 1:5000, Abcam) was added and incubated for 1 h at room temperature. The results were analyzed on the Image Quant LAS 4000C gel imager (GE, USA) [47].

### Flow cytometry

THP-1 cells and peritoneal macrophages from nude mice were collected and fixed overnight at 4 °C in 1% paraformaldehyde (PFA) after being washed with PBS. After overnight incubation, the cells were rewashed and resuspended in flow cytometry buffer (1 × PBS buffer containing 1% FSA) and incubated with CD163 (#562643, BD Biosciences, USA) and CD11B (#562399, BD Biosciences, USA) antibodies at 20 °C for 30 min. Finally, the cells were washed, resuspended, and analyzed using a flow cytometer (BD Biosciences, USA) following the manufacturer's instructions [54].

### Nude mouse xenograft model

Thirty 6-week-old BALB/c nude mice (Beijing Institute of Pharmacology, Chinese Academy of Medical Sciences) were kept in separate cages. Nude mice were randomized into 3 groups (10 in each group): (1) sh-NC + oe-NC group, in which mice were subcutaneously injected with sh-NC + oe-NC transfected 786-O cells, (2) sh-AGAP2-AS1 + oe-NC group, in which mice were subcutaneously injected with sh-AGAP2-AS1 + oe-NC transfected 786-O cells, and (3) sh-AGAP2-AS1 + oe-THBS2 group, in which mice were subcutaneously injected with 786-O cells transfected with sh-AGAP2-AS1 + oe-THBS2. All injections were performed on the dorsal skin. The tumor size was measured every day, and the survival time of nude mice was recorded. After 6 weeks, the mice were euthanized. The tumor was removed and weighed. Tumor volume was calculated as π(a^2^b)/6, where a indicates tumor width and b indicates tumor length.

### Immunohistochemistry

The ccRCC tissues were paraffin-embedded and cut into sections subjected to antigen retrieval. After blockage using normal goat serum (Sangong, Shanghai, China) for 20 min at room temperature, the samples were immunostained with mouse anti-Ki67 (ab8191, 1:50, Abcam) primary antibody and secondary antibody goat anti-nude mouse IgG (ab205719, 1:5000, Abcam). Finally, the results were observed under an upright microscope (BX63, Olympus, Japan) [48].

### H&E staining

The tumors and lung tissues from different groups of nude mice were collected and fixed in 10% neutral formalin. They were then embedded in paraffin and dewaxed with xylene. Subsequently, the tumor tissues were sectioned, stained with hematoxylin and eosin, rinsed with distilled water, immersed in 95% ethanol, stained with eosin Y, hydrated with gradient ethanol, dehydrated with xylene, air-dried, and observed under an optical microscope [55].

### Statistical analysis

All data were analyzed by SPSS 21.0 and expressed as mean ± standard deviation. First, a normality test was performed. If the data obeyed a normal distribution, a paired t-test was used between the ccRCC and para-cancerous tissue. The independent sample *t*-test was used for analysis between the two groups. One-way analysis of variance (ANOVA) and Tukey's post-hoc test was used to compare multiple groups. Data comparison between groups at different time points was performed by repeated measures ANOVA followed by Tukey's post-hoc test. A nonparametric test was used when the data did not show normal distribution. *p* < 0.05 indicates statistically significant.

## Results

RNA methylation has been reported to play an essential role in RCC, and m6A is one of the most abundant and prevalent post-transcriptional modifications. In this study, we aimed to find the m6A regulators and their specific molecular mechanisms in RCC growth. The bioinformatics analysis revealed that the m6A regulator IGF2BP3 was highly expressed in RCC, which could stabilize AGAP2-AS1 expression through m6A modification. It was also found that AGAP2-AS1 upregulated THBS2 expression by competitively binding to the downstream target miR-9-5p and inhibiting its expression, which in turn activated the PI3K/AKT signaling pathway, thereby inducing macrophage M2 polarization and promoting RCC development and progression.

### IGF2BP3 stabilizes AGAP2-AS1 through m6A modification in ccRCC cells

**Fig. 1:**
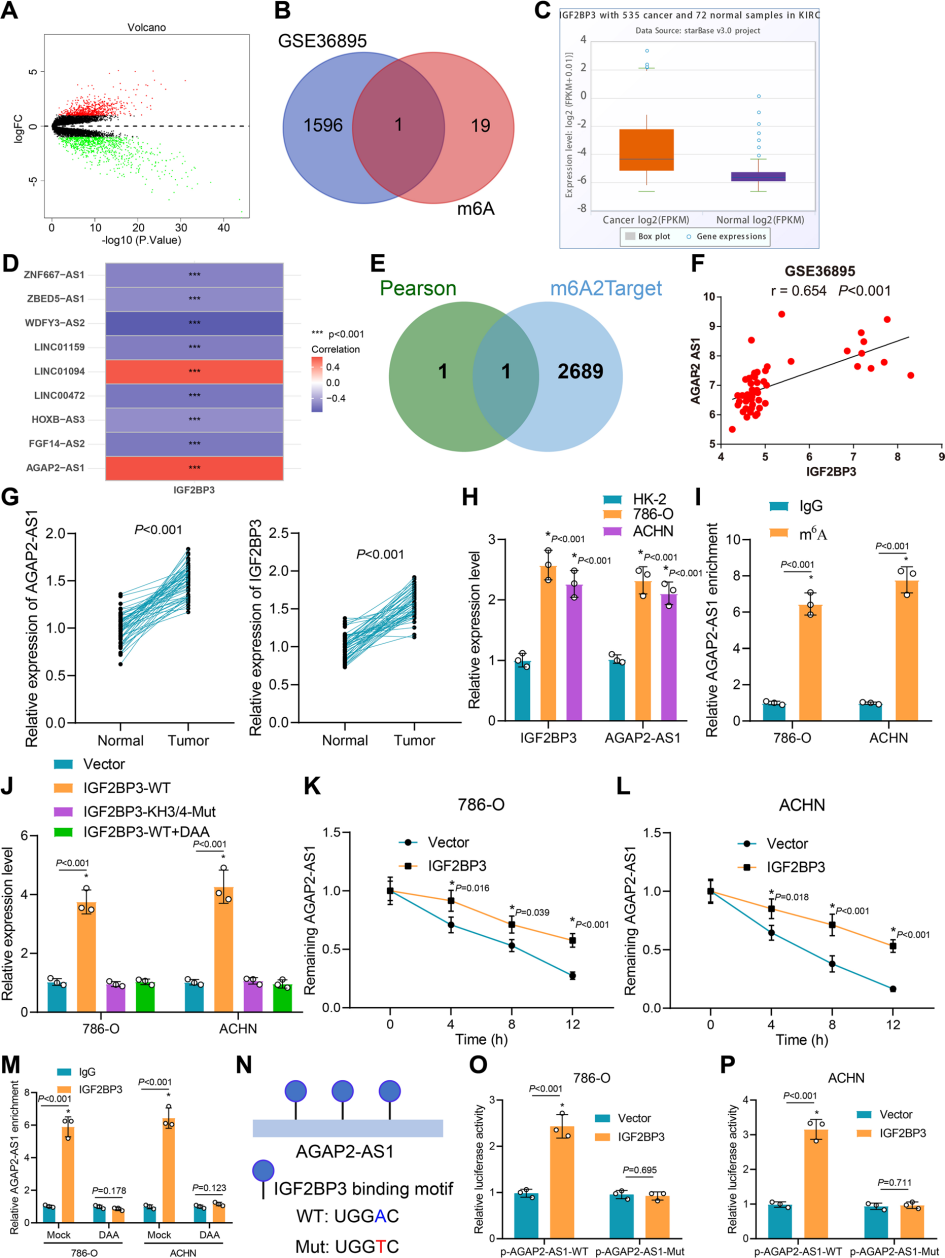
IGF2BP3 affects the expression of AGAP2-AS1 in RCC through m6A modification. **A** Volcano maps of normal kidney tissue and RCC samples in the GSE36895 dataset. Red dots refer to upregulated genes, green dots refer to downregulated genes, and black dots refer to genes without significant differential expression. **B** Venn diagram of differentially expressed genes and m6A regulators. **C** Expression of IGF2BP3 in RCC. **D** Heat map of correlation between IGF2BP3 and differentially expressed lncRNAs in the GSE36895 dataset. Red indicates a positive correlation, and blue indicates a negative correlation. **E** Venn map of the overlap of AGAP2-AS1 and LINC01094, which are positively related to IGF2BP3, with IGF2BP3 substrates in m6a2target database. **F** Correlation between IGF2BP3 and AGAP2-AS1 expression. **G** The levels of AGAP2-AS1 and IGF2BP3 in clinical samples. **H** Expression of AGAP2-AS1 in ccRCC cells. **I** meRIP detected the modification of m6A on AGAP2-AS1. **J** AGAP2-AS1 expression in cells; **K**, **L** Half-life of AGAP2-AS1 after actinomycin D treatment and IGF2BP3 overexpression. **M** AGAP2-AS1 enrichment in cells tested by RIP. **N** Schematic diagram of the binding sites of AGAP2-AS1 and IGF2BP3. **O**, **P** Luciferase activity in different treatment groups. * p < 0.05 vs. Compared with Normal group, HK-2 group, IgG group or Vector group

In total, 726 upregulated genes and 871 downregulated genes were identified from GSE36895 (Fig. 1A). Additionally, 20 widely recognized m6A regulators were obtained from published studies [23, 25, 32]. Venn diagram depicted that IGF2BP3 was the only intersected gene among the differentially expressed genes from GSE36895 and the 20 m6A regulators (Fig. 1B). In addition, we further analyzed the TCGA data in the ENCORI database and found that IGF2BP3 was highly expressed in ccRCC (Fig. 1C). Moreover, we screened the differentially expressed lncRNAs positively correlated with IGF2BP3 from GSE36895 by Pearson analysis with |cor|> 0.5 and P value < 0.05. The findings showed that AGAP2-AS1 and LINC01094 were positively correlated with IGF2BP3 (Fig. 1D), which were further intersected with IGF2BP3 substrates from the m6A2Target database. AGAP2-AS1 was the substrate of IGF2BP3 (Fig. 1E, F). It has been reported that AGAP2-AS1 is associated with poor survival and prognosis in RCC patients [17, 18]. Thus, IGF2BP3 could stabilize AGAP2-AS1 through m6A modification in RCC.

The expression of AGAP2-AS1 and IGF2BP3 was first detected in 50 clinical samples of ccRCC. The results showed that, compared with para-cancerous tissues, the expression of AGAP2-AS1 (t = 15.03, p < 0.001) and IGF2BP3 (t = 15.58, p < 0.001) increased significantly in ccRCC tissues (Fig. 1G). Their expressions were further verified in cell lines, which showed that compared with HK-2 cells, the levels of IGF2BP3 (p < 0.001) and AGAP2-AS1 (F = 48.38, p < 0.001) were significantly higher expressed in ccRCC cell lines of 786-O and ACHN (Fig. 1H). In addition, the merit assay demonstrated that compared with the IgG antibody, the m6A antibody significantly enriched AGAP2-AS1 (t = 15.25, p < 0.001; t = 16.33, p < 0.001) (Fig. 1I).

The KH3-4 domain is the core region where IGF2BP binds to m6A [56]. We constructed an IGF2BP3 plasmid with a mutation in the KH3-4 domain. PCR described that overexpression of IGF2BP3-WT significantly increased the expression of AGAP2-AS1 (p < 0.001), while overexpression of IGF2BP3-KH3/4-Mut did not affect AGAP2-AS1 (p = 0.974). There was no significant change in AGAP2-AS1 after overexpression of IGF2BP3 and the use of DAA (p = 0.972) (Fig. 1J). Overexpression of IGF2BP3 with KH3-4 domain mutations leads to an increase in IGF2BP3 expression, but it fails to exert its m6A binding function; hence, it does not affect the expression of AGAP2-AS1. These findings suggest that IGF2BP3 can regulate AGAP2-AS1 expression through m6A modifications.

Meanwhile, the half-life test results showed that overexpression of IGF2BP3-WT significantly prolonged the half-life of AGAP2-AS1 in 786-O (Fig. 1K) and ACHN cells (p < 0.001) (Fig. 1L). Furthermore, RIP eleborated that IGF2BP3 antibody enriched AGAP2-AS1 (t = 13.71, p < 0.001; t = 14.86, p < 0.001), while DAA treatment eliminated this effect (t = 1.631, p = 0.178; t = 1.950, p = 0.123) (Fig. 1M).

Through sequence alignment of AGAP2-AS1, two IGF2BP3 binding sites (TGGAC) were identified. Subsequently, the adenine base in the common m6A modification site was replaced with thymine (T) to abolish the m6A modification (Fig. 1N). Corresponding luciferase reporter vectors were constructed, and dual-luciferase reporter gene assays were conducted. The results demonstrate that overexpression of IGF2BP3 significantly enhances the activity of AGAP2-AS1-WT luciferase, while the activity of AGAP2-AS1-MUT luciferase remains unchanged (Fig. 1O, P). These experiments suggest that IGF2BP3 can stabilize AGAP2-AS1 expression through m6A modification in RCC cells.

Taken together, IGF2BP3 stabilizes the expression of AGAP2-AS1 through m6A modification in ccRCC cells.

### AGAP2-AS1 promotes the malignant properties of ccRCC cells and induces the polarization of macrophages to M2

**Fig. 2:**
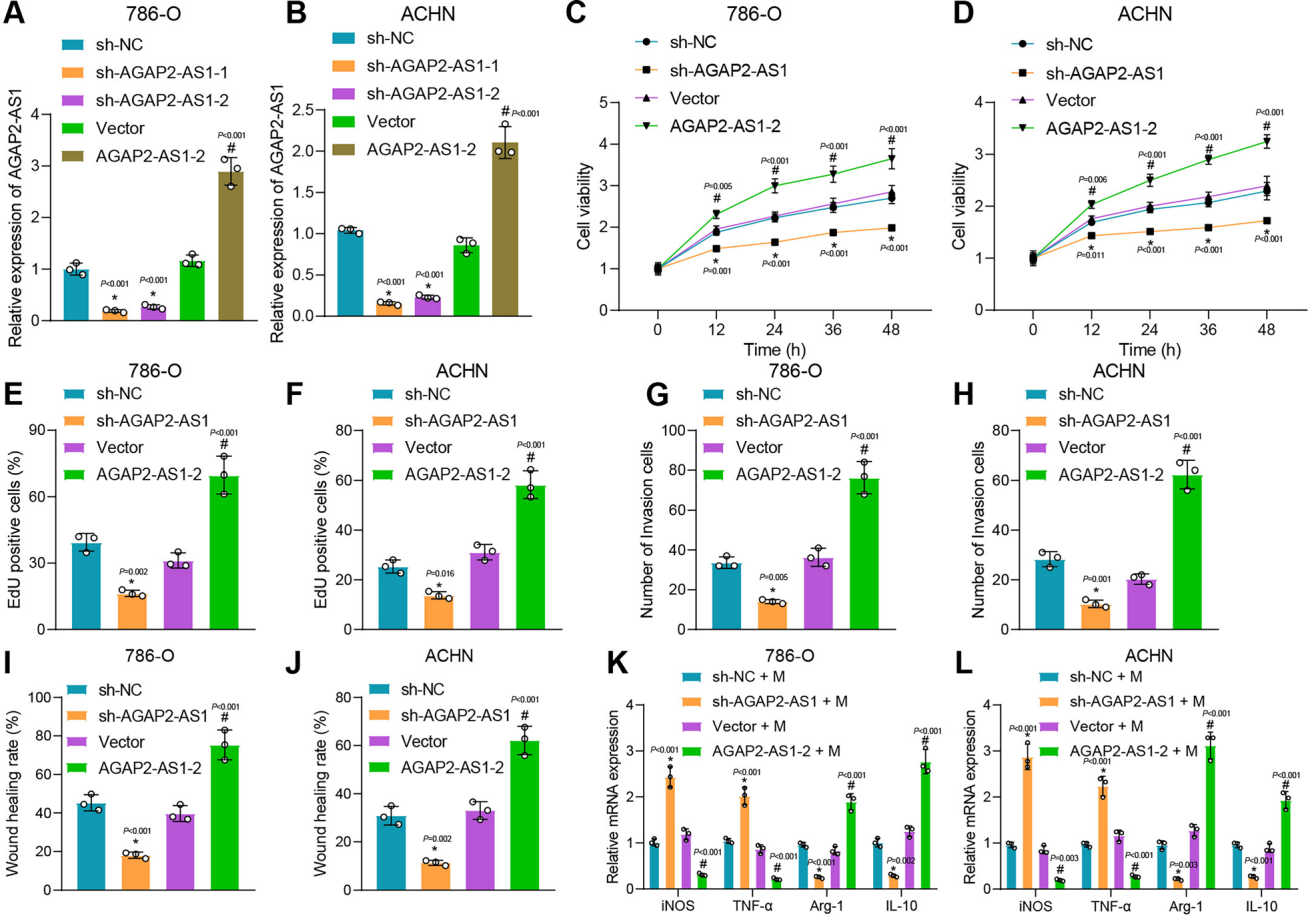
AGAP2-AS1 induces RCC cell proliferation, invasion and migration, and macrophage polarization. **A**, **B** Transfection efficiency of sh-AGAP2-AS1 by RT-qPCR. **C**, **D** Cell viability test by CCK-8. **E**, **F** Cell proliferation results by EdU assay. **G**, **H** Cell invasion results by Transwell assay. **I**,** J**  Cell migration results by wound healing assay. **K**, **L** The expression levels of M1 and M2 polarization markers in macrophages. * p < 0.05 *vs*. sh-NC group or sh-NC + M group. # p < 0.05 *vs*. Vector group or Vector + M group. In the groups, M refers to PMA + THP-1

Based on the bioinformatics above analysis, it is revealed that IGF2BP3 and AGAP2-AS1 are both highly expressed in both RCC cancer tissues and cells. Additionally, it has been observed that IGF2BP3 stabilizes the expression of AGAP2-AS1 in RCC cells through m6A modification. We then focused on the critical role of AGAP2-AS1 in ccRCC cell functions. As displayed by PCR, sh-AGAP2-AS1-1 (p < 0.001) had a higher knockdown efficiency (Fig. 2A, B), and thus sh-AGAP2-AS1-1 was used for further analysis. CCK-8 assay revealed that the cell viability was significantly reduced after silencing AGAP2-AS1 (p < 0.001); however, overexpression of AGAP2-AS1 led to opposing tendencies (p < 0.001) (Fig. 2C, D). EdU cell proliferation assay showed that the number of EdU-positive cells was significantly reduced after silencing AGAP2-AS1 (p < 0.001), while that was significantly increased after overexpression of AGAP2-AS1 (p < 0.001) (Fig. 2E, F). Transwell invasion assay and wound healing assay also demonstrated that the ability of cell invasion and migration was reduced after silencing AGAP2-AS1 (p < 0.01), while it was significantly increased after overexpression of AGAP2-AS1 (p < 0.001) (Fig. 2G–J).

Recent studies have shown that macrophage polarization towards the M2 phenotype is associated with a poorer clinical prognosis in RCC patients and promotes cancer progression [57]. M2-polarized macrophages stimulate cellular proliferation, extracellular matrix synthesis, and tissue remodeling, facilitating tumor progression [58, 59]. Therefore, our investigation aims to determine whether AGAP2-AS1 can induce M2 polarization in macrophages. In addition, PMA was used to induce THP-1 to differentiate into M0 macrophages, followed by co-culture with treated ccRCC cells. RT-qPCR showed that the expression of M1 polarization markers *iNOS* (p < 0.01) and *TNF-α* (p < 0.001) increased significantly after being co-cultured with the supernatant from the AGAP2-AS1 knockdown ccRCC cells, while M2 polarization markers of *Arg-1* (p < 0.01) and *IL-10* (p < 0.01) was significantly reduced (Fig. 2K, L). On the contrary, an opposing tendency was witnessed after co-culture with supernatant from ccRCC cells overexpressing AGAP2-AS1 (Fig. 2K, L). Similarly, flow cytometry showed that the number of CD163^+^ macrophages increased after co-culture with the supernatant of RCC cells overexpressing AGAP2-AS1, while co-culture of the supernatant from the AGAP2-AS1 knockdown ccRCC cells with M0 macrophages brought about opposite finding (Additional file 1: Figure S1A and B). These indicate that AGAP2-AS1 promotes the polarization of M0 macrophages to M2.

### AGAP2-AS1 up-regulates THBS2 expression by binding to miR-9-5p

**Fig. 3:**
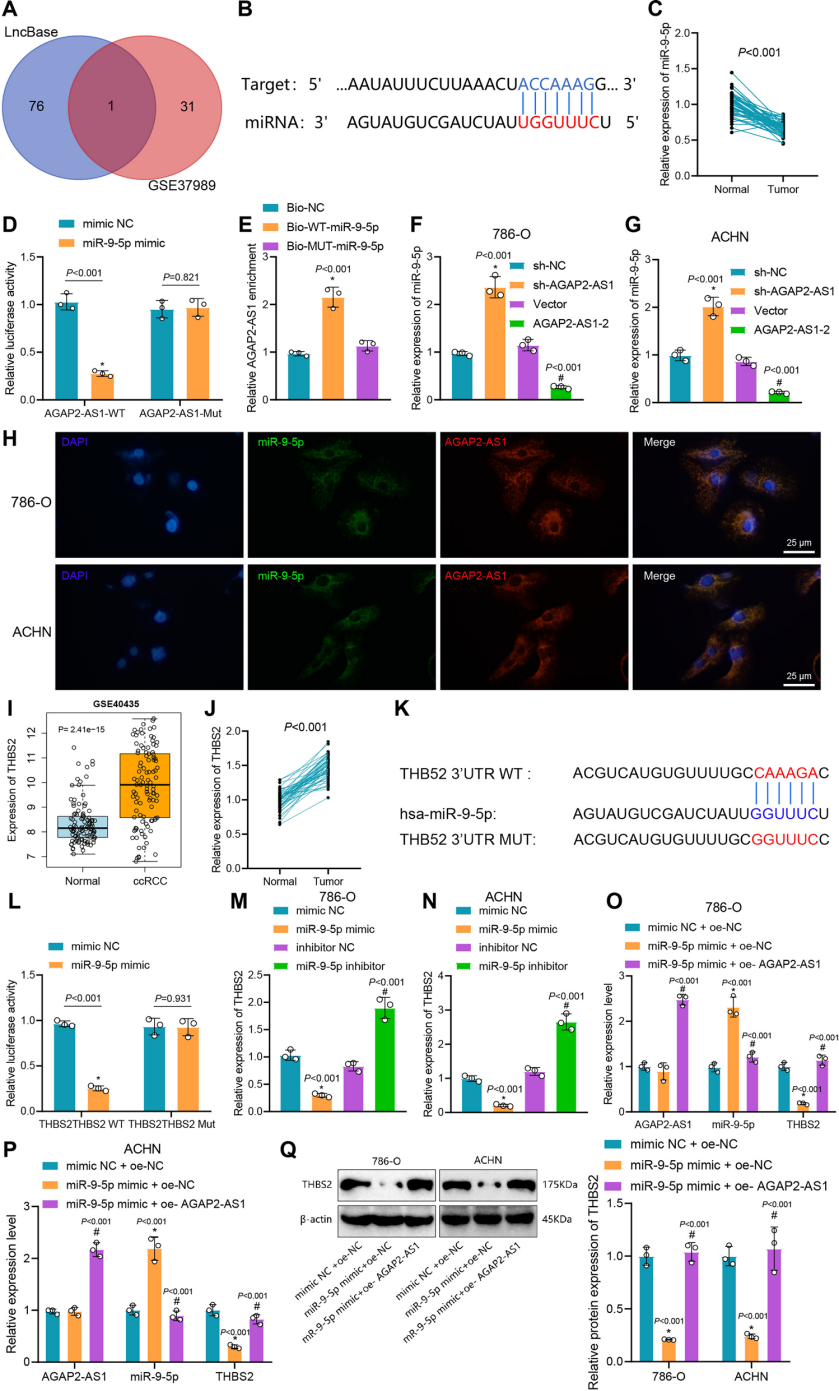
AGAP2-AS1 binds to miR-9-5p to elevate THBS2 expression. **A** Venn diagram showing the overlap of LncBase database predicted AGAP2-AS1-bound miRNAs with lowly expressed miRNAs in the GSE37989 dataset. **B** Schematic diagram of the binding site between AGAP2-AS1 and miR-9-5p. **C** miR-9-5p expression in clinical samples. **D**, **E** Binding of AGAP2-AS1 and miR-9-5p assessed by dual luciferase reporter assay. **F**, **G** Expression of miR-9-5p in 786-O and ACHN cells after AGAP2-AS1 silencing or overexpression. **H** FISH analysis showing the co-localization of AGAP2-AS1 and miR-9-5p in 786-O and ACHN cells. **I** The expression of THBS2 in ccRCC in the GSE40435 dataset. **J** The expression of THBS2 in clinical samples. **K** Schematic diagram of the binding site between miR-9-5p and THBS2. **L** Binding of miR-9-5p and THBS2 assessed by dual luciferase reporter assay. **M**, **N** THBS2 expression in cells following miR-9-5p inhibitor/mimic treatment. **O**, **P** Expression of AGAP2-AS1, miR-9-5p and THBS2 in cells following miR-9-5p mimic and oe-AGAP2-AS1 treatment. **Q** THBS2 protein expression following miR-9-5p mimic and oe-AGAP2-AS1 treatment. * p < 0.05 vs. Normal group, mimic NC group, Bio-NC group, sh-NC group or mimic NC + oe-NC group. # p < 0.05 *vs*. Vector group or miR-9-5p mimic + oe-NC group

LncRNAs function mainly by affecting the expression of downstream miRNAs [60]. We then predicted the miRNAs that bind to AGAP2-AS1 through the LncBase database and intersected them with the low-expressed miRNAs in the GSE37989 dataset. Finally, we screened out miR-9-5p as the target of AGAP2-AS1 (Fig. 3A). In addition, the binding site of AGAP2-AS1 with miR-9-5p was predicted using the TargetScan database (Fig. 3B). The expression of miR-9-5p in 50 ccRCC tissues was significantly reduced compared with that in para-cancerous tissues (t = 12.46, p < 0.001) (Fig. 3C). Luciferase assay showed that compared with mimic NC, miR-9-5p mimic significantly reduced the luciferase activity of AGAP2-AS1-WT (t = 14.41, p < 0.001), but there was no significant difference for AGAP2-AS1-MUT (t = 0.2415, p = 0.821) (Fig. 3D). RNA pull-down results showed that compared with Bio-MUT-miR-9-5p and Bio-NC groups, AGAP2-AS1 bound to Bio-WT-miR-9-5p increased (p < 0.001) (Fig. 3E). These results indicate that AGAP2-AS1 can bind miR-9-5p.

Furthermore, RT-qPCR determination of miR-9-5p expression showed that the expression of miR-9-5p was increased after silencing AGAP2-AS1 (p < 0.001), while the expression of miR-9-5p was decreased after AGAP2-AS1 overexpression (p < 0.001) (Fig. 3F, G). Meanwhile, FISH displayed that AGAP2-AS1 and miR-9-5p co-localized in cells (Fig. 3H).

Finally, we predicted the target genes of miR-9-5p through the TargetScan database and performed KEGG analysis. The results showed that the target genes of miR-9-5p were enriched in the PI3K-Akt signaling pathway (Additional file 2: Figure S2). Previous studies have revealed that THBS2 gene silencing inhibits the PI3K-Akt signaling pathway, which can affect macrophage polarization [55, 61]. Here, by GSE40435 dataset analysis, we found that THBS2 was highly expressed in ccRCC tissues (Fig. 3I). The high expression of THBS2 was further verified in clinical samples of ccRCC (t = 16.35, p < 0.001) (Fig. 3J).

The binding site between miR-9-5p and THBS2 was predicted by the TargetScan (Fig. 3K). Luciferase assay showed that miR-9-5p mimic reduced the luciferase activity of THBS2-WT (t = 29.88, p < 0.001), but for THBS2-MUT, there was no significant difference (t = 0.09252, P = 0.931) (Fig. 3L). Detection of THBS2 expression by RT-qPCR clarified that the expression of THBS2 was significantly reduced following miR-9-5p mimic treatment while elevated upon miR-9-5p inhibitor (p < 0.001) (Fig. 3M, N).

Next, we conducted a combined intervention of AGAP2-AS1 and miR-9-5p to examine THBS2 expression. The results showed that, compared to the mimic NC + oe-NC group, there was no significant difference in AGAP2-AS1 expression in the miR-9-5p mimic + oe-NC group, but an increase in miR-9-5p levels and a significant decrease in THBS2 expression. Additionally, compared to the miR-9-5p mimic + oe-NC group, the miR-9-5p mimic + oe-AGAP2-AS1 group exhibited a decrease in miR-9-5p levels and a significant increase in AGAP2-AS1 and THBS2 expression (Fig. 3O, P). Western blot analysis confirmed the consistency of THBS2 expression with the RT-qPCR results (Fig. 3Q). These results demonstrate that AGAP2-AS1 can upregulate THBS2 expression by competitively binding to miR-9-5p.

Collectively, AGAP2-AS1 up-regulates the expression of THBS2 by competitively binding to miR-9-5p.

### THBS2 promotes M2 polarization of macrophages by activating the PI3K/Akt pathway

**Fig. 4:**
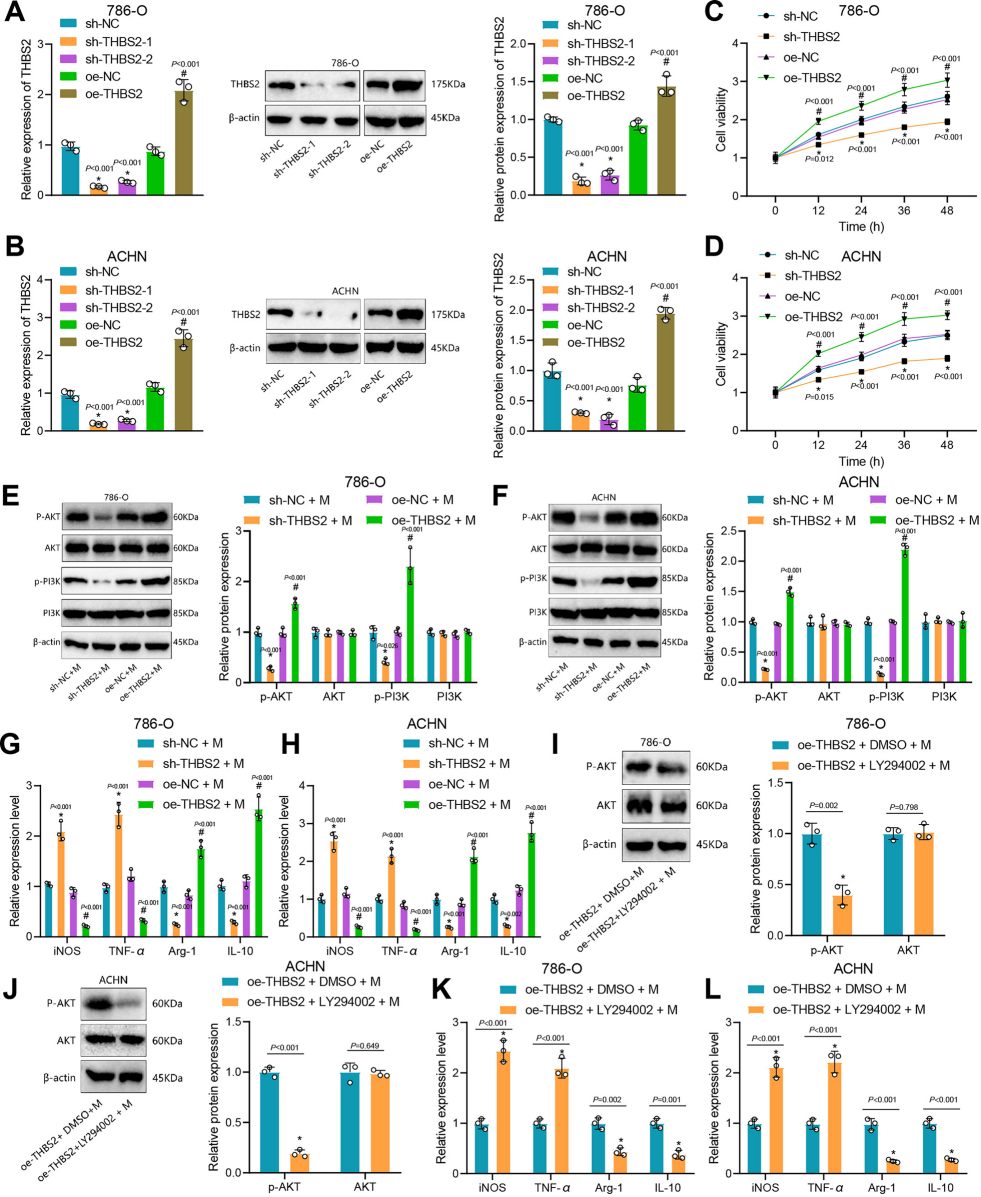
THBS2 activates the PI3K/Akt signaling pathway to induce M2 polarization of macrophages. **A**, **B** Transfection efficiency of sh-THBS2 detected by RT-qPCR. **C**, **D** Cell viability test by CCK-8. **E**, **F** Expression of PI3K/AKT signaling pathway proteins after co-culture of sh-THBS2-/oe-THBS2-treated cells with macrophages. **G**, **H** Expression of M1 polarization and M2 polarization markers in macrophages. **I**, **J** Expression of PI3K/AKT signaling pathway proteins. **K**, **L** Macrophage M1 and M2 polarization marker expression. * p < 0.05 *vs*. sh-NC group, sh-NC + M group or oe-THBS2 + DMSO + M group. # p < 0.05 *vs*. oe-NC group or oe-NC + M group

According to the literature, silencing the THBS2 gene can inhibit the PI3K-Akt signaling pathway. Conversely, activating the PI3K/AKT signaling pathway induces M2 macrophage polarization, thereby promoting cancer cell growth [54, 61]. We further silenced THBS2 expression in ccRCC cells. The sh-THBS2-1 had a better silencing efficiency (Fig. 4A, B) and thus was used in further analysis. CCK-8 assay showed that cell viability decreased significantly after silencing THBS2 but increased significantly after overexpression of THBS2 (Fig. 4C, D). In addition, after co-culturing THBS2 knockdown ccRCC cells with macrophages, we used Western blot to detect the expression of PI3K/AKT signaling pathway-related proteins in macrophages. The results showed that the expression of p-PI3K and p-AKT in the sh-THBS2 + M (PMA + THP-1) group was significantly reduced compared with the sh-NC + M group (Fig. 4E, F). Compared with the oe-NC + M group, the expression of p-PI3K and p-AKT in the oe-THBS2 + M group increased significantly (Fig. 4E, F). Moreover, compared with the sh-NC + M group, the expression of *iNOS* and *TNF-*α in the sh-THBS2 + M group increased, while the expression of *Arg-1* and *IL-10* reduced (Fig. 4G, H). In the oe-NC + M group, the expression of *iNOS* and *TNF-α* in the oe-THBS2 + M group was reduced, whereas the expression of *Arg-1* and *IL-10* was increased (Fig. 4G, H). The above results indicate that THBS2 may affect the M1/M2 polarization of macrophages.

To further explore whether THBS2 affects the polarization of macrophages by affecting the PI3K/AKT signaling pathway, we used the PI3K/AKT inhibitor LY294002. Compared with the oe-THBS2 + DMSO + M group, the expression of p-AKT (t = 7.40, p = 0.002; t = 5.62, p = 0.005) in the oe-THBS2 + LY294002 + M group reduced (Fig. 4I, J). For analysis of M1/M2 polarization, RT-qPCR showed that in oe-THBS2 + LY294002 + M group, *iNOS* (t = 10.59, p < 0.001; t = 8.92, p < 0.001) and *TNF-α* (t = 8.728, p < 0.001; t = 8.998, p < 0.001) levels increased significantly, while *Arg-*1 (t = 7.08, p = 0.002; 10.73, p < 0.001) and *IL-10* (t = 8.46, p = 0.001; t = 12.53, p < 0.001) levels decreased significantly (Fig. 4K, L). Meanwhile, flow cytometry showed that compared with the oe-THBS2 + DMSO group, the number of CD163^+^ cells in the oe-THBS2 + LY294002 group was reduced (Additional file 1: Figure S1C and D). These results suggest that THBS2 may promote M2 polarization of macrophages by activating the PI3K/Akt signaling pathway.

### AGAP2-AS1/miR-9-5p/THBS2 induces M2 polarization of macrophages and promotes the malignant behaviors of ccRCC cells

**Fig. 5:**
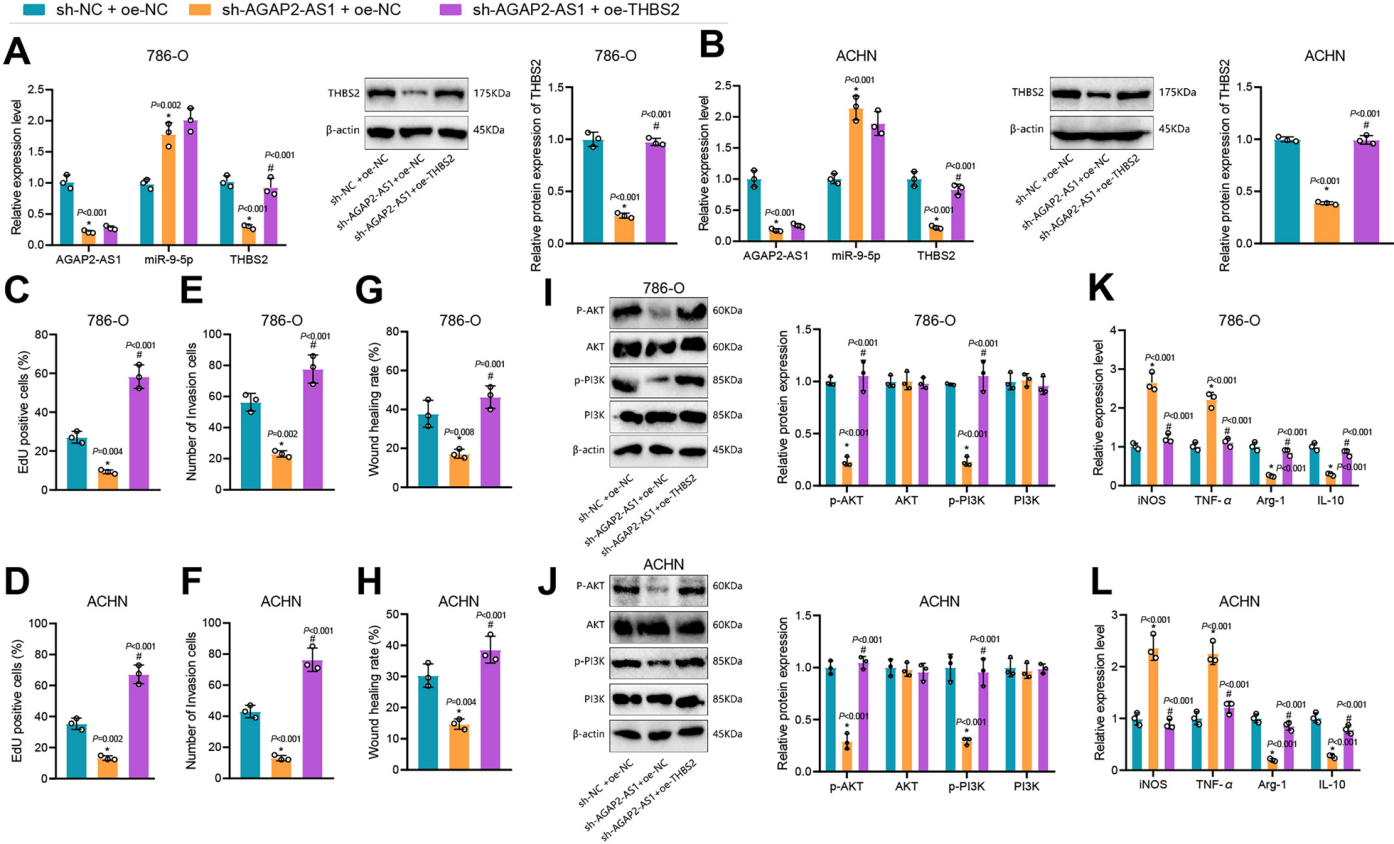
AGAP2-AS1/miR-9-5p/THBS2 axis facilitates the malignant characteristics of ccRCC cells and M2 polarization of macrophages. **A**, **B** AGAP2-AS1, miR-9-5p and THBS2 expression in each group of cells following sh-AGAP2-AS1 and oe-THBS2 treatment. **C**, **D** Cell proliferation ability by EdU assay. **E**, **F** Cell invasion ability. **G**, **H** Cell migration ability. **I**, **J** Expression of PI3K/AKT signaling pathway proteins. **K**, **L** Expression of macrophage M1 and M2 polarization markers. * p < 0.05 *vs*. sh-NC + oe-NC group. # p < 0.05 *vs*. sh-AGAP2-AS1 + oe-NC group

We knocked down AGAP2-AS1 and overexpressed THBS2 in ccRCC cells to further verify the role of the AGAP2-AS1/miR-9-5p/THBS2 axis in the biological functions of ccRCC cells. We found that the expression of AGAP2-AS1 and THBS2 was reduced, and that of miR-9-5p was increased following sh-AGAP2-AS1 treatment, while additional oe-THBS2 caused opposing tendency (Fig. 5A, B).

Cell proliferation by EdU assay showed that sh-AGAP2-AS1 treatment reduced cell proliferation ability, while additional oe-THBS2 caused an enhanced one (Fig. 5C, D). Furthermore, the cell invasion and migration abilities after sh-AGAP2-AS1 treatment were reduced, while those were enhanced following further oe-THBS2 treatment (Fig. 5E–H).

Next, we co-cultured macrophages with ccRCC cells with AGAP2-AS1 knockdown and THBS2 overexpression. After that, changes in PI3K/Akt pathway essential proteins and macrophage polarization were analyzed. As shown in Fig. 5I and J, compared to the sh-NC + oe-NC + M group, the expression of p-PI3K and p-AKTin the sh-AGAP2-AS1 + oe-NC + M group was significantly reduced. Their levels significantly increased in the sh-AGAP2-AS1 + oe-THBS2 + M group than in the sh-AGAP2-AS1 + oe-NC + M group. Analysis of macrophage polarization markers showed that the expressions of *iNOS* and *TNF-α* increased while those of *Arg-*1 and *IL-10* decreased in the sh-AGAP2-AS1 + oe-NC + M group than the sh-NC + oe-NC + M group (Fig. 5K, L). CD163 is a marker of M2 macrophages [62, 63]. Compared with the sh-AGAP2-AS1 + oe-NC + M group, the expression of *iNOS* and *TNF-α* in the sh-AGAP2-AS1 + oe-THBS2 + M group was significantly reduced, but the *Arg-1* and *IL-10* expression increased (Fig. 5K, L). Moreover, flow cytometry results depicted that the number of CD163^+^ cells in the sh-AGAP2-AS1 + oe-NC + M group was lower than that in the sh-NC + oe-NC + M group. Compared with the sh-AGAP2-AS1 + oe-NC + M group, the number of CD163^+^ cells in the sh-AGAP2-AS1 + oe-THBS2 + M group was increased (Additional file 1: Figure S1E and F).

The AGAP2-AS1/miR-9-5p/THBS2 axis may induce M2 polarization of macrophages and promote the development of ccRCC cells.

### *AGAP2-AS1/miR-9-5p/THBS2 axis induces M2 polarization of macrophages and promotes the development of ccRCC *in vivo

**Fig. 6:**
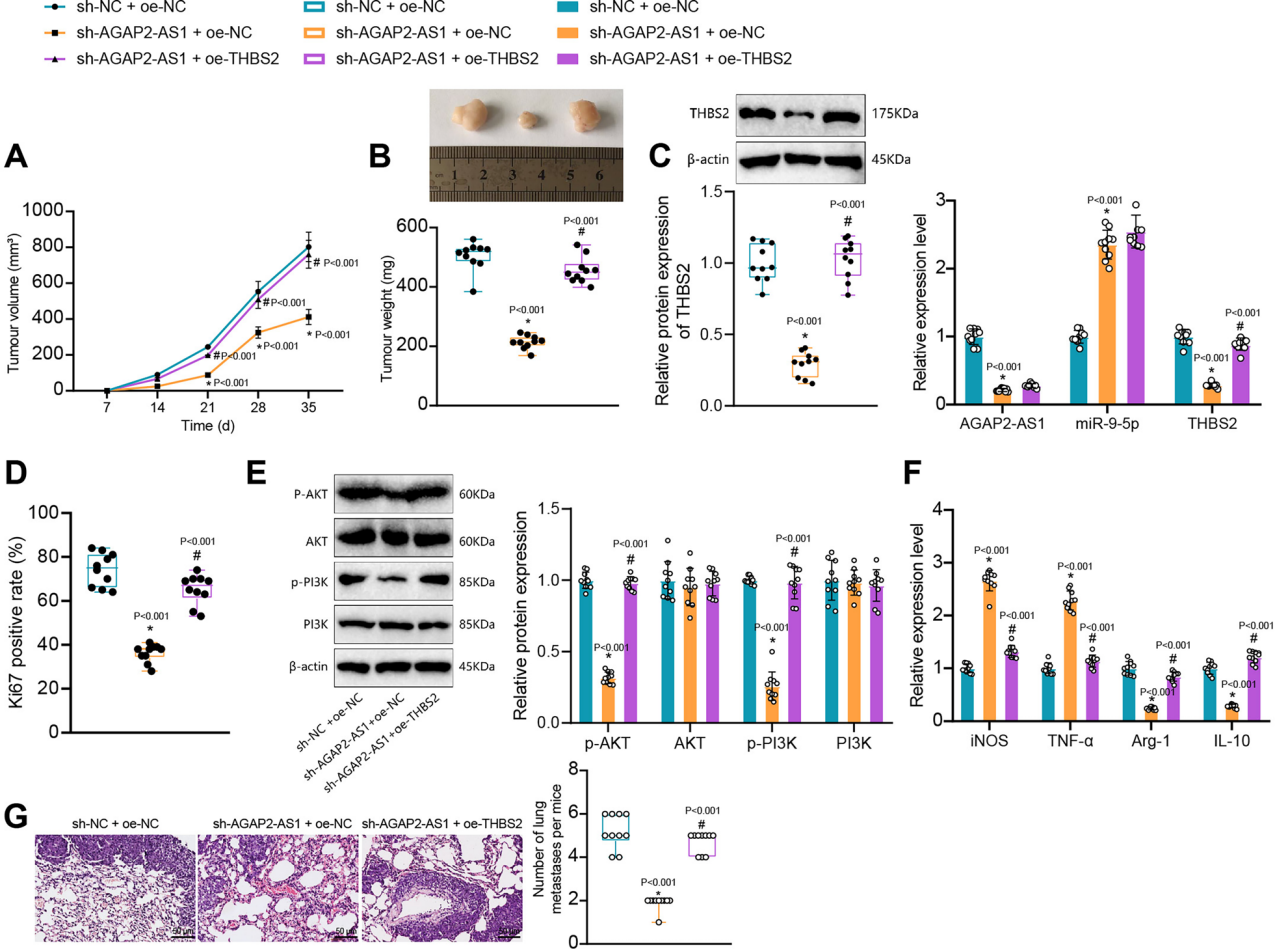
AGAP2-AS1/miR-9-5p/THBS2 axis induces M2 polarization of macrophages and aggravates RCC in vivo. **A** Tumor volume following sh-AGAP2-AS1 and oe-THBS2 treatment. **B** Tumor weight following sh-AGAP2-AS1 and oe-THBS2 treatment. **C** AGAP2-AS1, miR-9-5p and THBS2 expression in tumors of each group following sh-AGAP2-AS1 and oe-THBS2 treatment. **D** Ki67 expression level in tumor sections assessed by immunohistochemistry. **E** PI3K/AKT signaling pathway-related protein expression in tumors following sh-AGAP2-AS1 and oe-THBS2 treatment. **F** Macrophage M1 and M2 polarization marker expression levels tested by RT-qPCR. **G** Representative HE staining images of lung tissues of nude mice in each group and quantitative analysis of metastatic nodes in lung sections. * p < 0.05 *vs*. sh-NC + oe-NC group; # p < 0.05 *vs*. sh-AGAP2-AS1 + oe-NC group

After intervention, ACHN cells were inoculated into nude mice to establish the xenograft model. As shown in Fig. 6A, B, the tumor volume and weight of mice bearing sh-AGAP2-AS1-treated ACHN cells were significantly reduced; however, those were increased following further oe-THBS2 treatment. Moreover, analysis of the expression of AGAP2-AS1, miR-9-5p and THBS2 in the tumor depicted that mice bearing sh-AGAP2-AS1-treated ACHN cells showed reduced expression of AGAP2-AS1 and elevated expression of miR-9-5p; however, THBS2 expression was restored in the following further oe-THBS2 treatment (Fig. 6C). Immunohistochemistry revealed that the Ki67-positive cells in mice bearing sh-AGAP2-AS1-treated ACHN cells were significantly reduced while increased following further oe-THBS2 treatment (Fig. 6D).

Moreover, the expression of p-PI3K and p-AKT in mice bearing sh-AGAP2-AS1-treated ACHN cells was significantly reduced; however, opposing trends were seen following further oe-THBS2 treatment (Fig. 6E). As displayed by PCR, there were increased levels of *iNOS* and *TNF-α* while decreased levels of *Arg-1* and *IL-10* in tumors of mice bearing sh-AGAP2-AS1-treated ACHN cells, while the changes of these markers were reversed following further oe-THBS2 treatment (Fig. 6F). As revealed by flow cytometry, mice bearing sh-AGAP2-AS1-treated ACHN cells had fewer CD163^+^ cells, while mice bearing sh-AGAP2-AS1 + oe-THBS2-treated ACHN cells had increased CD163^+^ cells (Additional file 1: Figure S1G).

Finally, H&E staining showed that mice bearing sh-AGAP2-AS1-treated ACHN cells had fewer lung metastasis tumor nodules, while the tumor nodules metastasized to the lung tissue of nude mice bearing sh-AGAP2-AS1 + oe-THBS2-treated ACHN cells increased (Fig. 6G).

In summary, consistent with in vitro experiments, the AGAP2-AS1/miR-9-5p/THBS2 axis may also induce macrophage M2 polarization and promote the development of ccRCC in vivo.

## Discussion

It has been reported that m6A RNA modification regulates RNA metabolism and plays a vital role in the occurrence and development of many diseases, including cancer [64]. IGF2BPs are m6A-binding proteins, and if the m6A level of mRNA is reduced, the ability of IGF2BPs to bind to their targets will be attenuated [30]. In this study, through bioinformatics analysis, we found that IGF2BP3 was highly expressed in ccRCC. Besides, studies have shown that IGF2BP3 acts as an “m6A” reader to stabilize lncRNA [32, 64, 65]. Based on the prediction results of m6a2target and ccRCC dataset, we further screened the lncRNA AGAP2-AS1 that was positively correlated with IGF2BP3 in ccRCC. Two IGF2BP3 binding sites were identified on AGAP2-AS1. Meanwhile, we showed that IGF2BP3 stabilized the expression of AGAP2-AS1 through m6A modification in ccRCC cells. The findings suggested the potential of IGF2BP3-mediated AGAP2-AS1 in modulating the development of ccRCC. Hence, we conducted additional experiments to validate the role of IGF2BP3 and AGAP2-AS1 interaction in ccRCC.

Based on this study, we have gained insights into the activation of the PI3K-Akt pathway in RCC and its role in promoting RCC progression. This signaling pathway is regulated by various factors, as demonstrated in our study with the discovery of the AGAP2-AS1/miR-9-5p/THBS2 axis. Additionally, it is known that other factors influence this pathway. Previous research has shown that M2-EV inhibits NEDD4L through the transmission of miR-342-3p, thereby suppressing the ubiquitination and degradation of CEP55 by activating the PI3K/AKT/mTOR signaling pathway, which in turn promotes RCC cell growth, migration, and invasion [66]. Furthermore, viaGLUD1 inhibits kidney tumor occurrence and development by suppressing the PI3K/Akt/mTOR pathway [67], while LINC00460 promotes RCC development by affecting the PI3K/AKT pathway and decreasing the phosphorylation levels of AKT and mTOR [68].

Although studies have shown that AGAP2-AS1 may be a potential biomarker for early diagnosis and prognosis of various tumors, its role in kidney cancer, especially in ccRCC, is still unclear [17, 18]. The gene set enrichment analysis based on the TCGA database shows that the high expression of AGAP2-AS1 may be an independent predictor of the low survival rate of ccRCC patients [18], but experiments have not confirmed this. In this study, we found that AGAP2-AS1 was significantly up-regulated in cancer tissues and cell lines of ccRCC and that AGAP2-AS1 expression was significantly correlated with ccRCC tumor size, recurrence, and high histological grade phenotype (Additional file 3: Table S1). To explore the effect of AGAP2-AS1 on the biological functions of ccRCC cells, we constructed the AGAP2-AS1 knockdown cell line using lentivirus. Our results found that when AGAP2-AS1 was knocked down, cell viability was significantly reduced, the number of EdU-positive cells was significantly reduced, and the ability of cell invasion and migration was significantly reduced. These data indicate that AGAP2-AS1 promotes the malignant features of ccRCC cells. As a plastic and multifunctional cell population, Macrophages can exhibit different phenotypes under different physiological and pathological conditions, thus exerting different tumor-promoting or tumor-suppressing functions [69]. Alternatively, activated M2-type macrophages are mainly involved in anti-inflammatory response, which can promote tumor growth and invasion [70]. It is shown that M2-polarized macrophages can lead to a poorer clinical prognosis for ccRCC patients and promote cancer development [71]. Multiple studies have illustrated that lncRNAs can function as promoters or suppressors of M2-polarized macrophages to facilitate or delay the progression of human cancers such as hepatocellular carcinoma, endometrial, colorectal cancer, etc. [72–74]. Although AGAP2-AS1 has been proposed to be abundant in M2 macrophage-derived exosomes [75], whether AGAP2-AS1 also functions to affect the phenotype of macrophages in ccRCC remains unknown. Strikingly, our experiments suggested that AGAP2-AS1 induced the polarization of M0 macrophages to M2. This may be the mechanism underlying the promotive effect of AGAP2-AS1 on the proliferation, invasion, and migration of ccRCC cells.

Subsequently, the molecular mechanism underlying the tumor-promotive function of AGAP2-AS1 was to be explored. Competing endogenous RNAs (ceRNAs) exert crucial roles in post-transcriptional regulation of gene expression via a miRNA-related mechanism, contributing to their implication in cancer pathogenesis [76]. Recently, some lncRNAs have emerged as critical modulators of pro-tumorigenic or anti-tumorigenic genes by functioning as ceRNAs, such as H19, HOTAIR, etc. [76]. We predicted miR-9-5p as a miRNA bound by AGAP2-AS1 through the LncBase database. Further analysis in this study showed a co-localization of AGAP2-AS1 and miR-9-5p in cells. More and more evidence shows that miR-9-5p can affect the occurrence and development of tumors by regulating cell proliferation [43, 77]. The lncRNA embigin pseudogene 1-miR-9-5p axis dysregulation plays a vital role in the progression of RCC [43]. It is confirmed that miR-9-5p can predict the effective response of tyrosine kinase inhibitors in treating metastatic RCC and the progression-free survival after treatment [78]. Bioinformatics analysis found that miR-9-5p may be a potential oncogene of ccRCC [79]. In this study, we found that the expression of miR-9-5p was significantly reduced in tumor tissues of ccRCC patients, suggesting its potential significance in ccRCC. Additionally, the present study suggested that AGAP2-AS1 up-regulated the expression of THBS2 by competitively binding to miR-9-5p. Similarly, THBS2 was demonstrated as a direct target of miR-9 to confer miR-9-mediated role in glioma [80], yet this binding has not been mentioned in other cancers.

It is reported that PTPN3 may inhibit the progression of RCC through the PI3K/AKT signaling pathway [81]. Hofmann et al. found that the activation of the PI3K/AKT signaling pathway was related to the production of anti-inflammatory mediators and the polarization of macrophages [82]. Studies have shown that THBS2 gene silencing could inhibit the PI3K-AKT signaling pathway [55, 61]. THBS2 may be related to RCC metastasis and may be an independent prognostic factor for ccRCC patients [83]. Herein, our results revealed that THBS2 promoted M2 polarization of macrophages by activating the PI3K-AKT signaling pathway. When AGAP2-AS1 was knocked down, THBS2 was overexpressed, or AGAP2-AS1 was knocked down alone in ccRCC cells, the biological functions and macrophage phenotypes were assessed. We found that overexpression of THBS2 can reverse the tumor-suppressing effects caused by AGAP2-AS1 knockdown. These results indicated that the AGAP2-AS1/miR-9-5p/THBS2 axis promoted RCC development and macrophage M2 polarization by activating the PI3K/AKT signaling pathway. Finally, our in vivo xenograft model confirmed that the AGAP2-AS1/miR-9-5p/THBS2 axis induced the polarization of macrophage M2 and promoted the occurrence and development of ccRCC in nude mice.

## Conclusion

**Fig. 7:**
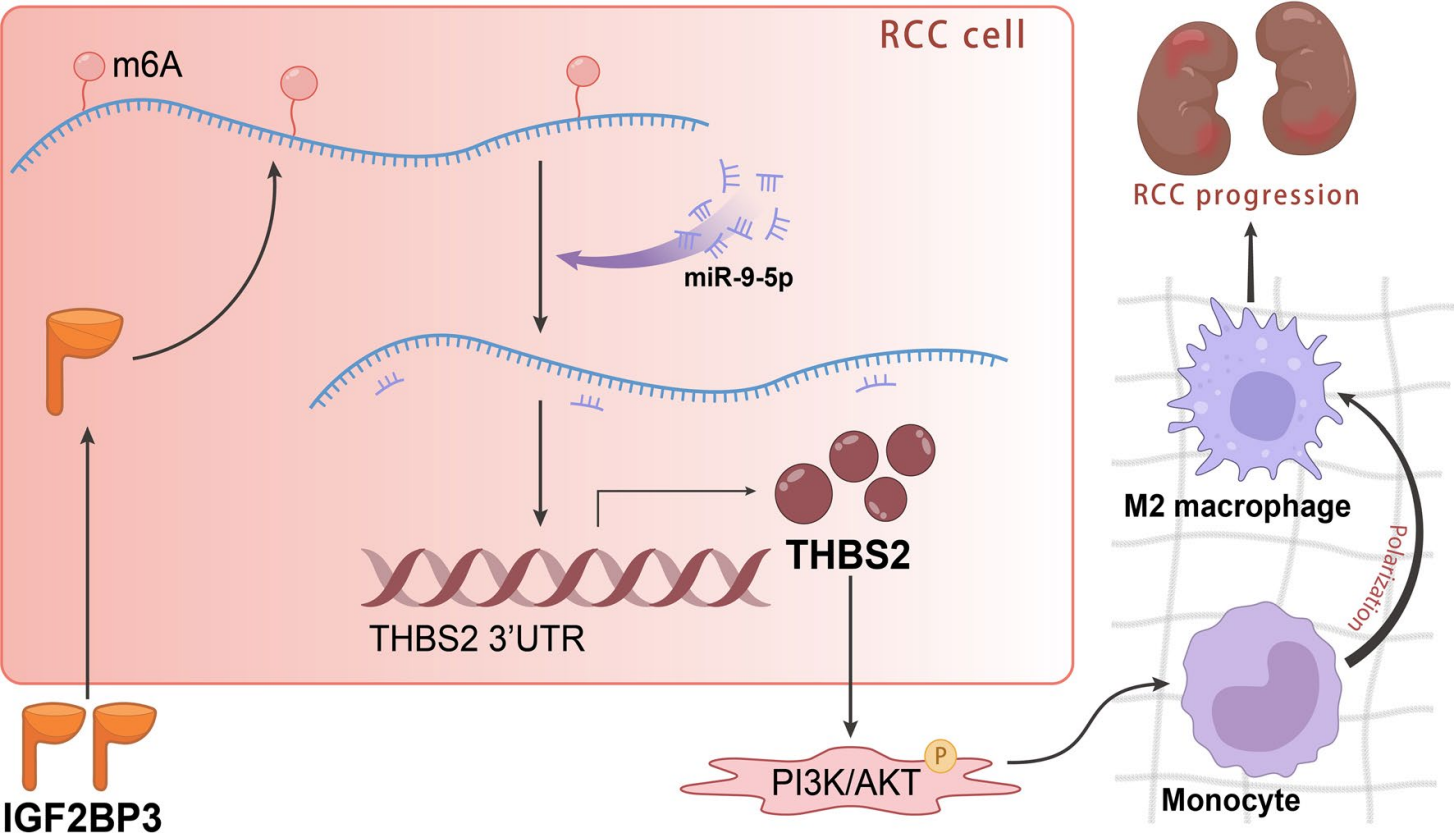
Schematic diagram of IGF2BP3/AGAP2-AS1/miR-9-5p/THBS2/PI3K-Akt axis in clear cell renal cell carcinoma. IGF2BP3 stabilizes AGAP2-AS1 through m6A modification and mediates the miR-9-5p/THBS2/PI3K-Akt signaling pathway to affect the M2 polarization of macrophages, thereby participating in renal cell carcinogenesis

In summary, our findings demonstrate that IGF2BP3 may stabilize the expression of AGAP2-AS1 through m6A modification, and up-regulate THBS2 by competitively binding to miR-9-5p, thereby activating the PI3K/AKT signaling pathway, and ultimately inducing the polarization of macrophages M2 and promoting the occurrence and development of ccRCC (Fig. 7). These findings offer valuable information for understanding the role of m6A modification in regulating lncRNA in the progression of ccRCC.

### Supplementary Information

**Additional file 1: Figure S1.** A and B: Flow cytometry results of macrophage M2 polarization marker CD163 expression following silencing or overexpression of AGAP2-AS1. C and D: Expression of M2 polarization marker CD163 in macrophages following flow cytometry treatment of oe-THBS2, DMSO, and LY294002. E and F: Expression of M2 polarization marker CD163 in macrophages co-cultured with sh-AGAP2-AS1-/oe-THBS2-treated cells by flow cytometry. G: Expression of M2 polarization marker CD163 in macrophages following sh-AGAP2-AS1 and oe-THBS2 treatment analyzed by flow cytometry.

**Additional file 2: Figure S2.** KEGG pathway enrichment analysis of miR-9-5p target genes. The top 15 results of the KEGG pathway enrichment analysis of miR-9-5p target genes are listed. The y-axis indicates pathways, and the X-axis indicates the ratio of genes enriched in the pathway

**Additional file 3: Table S1.** Clinical and pathological features of patients (N = 50). **Table S2.** RT-qPCR primer sequences.

## Data Availability

The datasets used and analyzed in the current study are attained from the corresponding author on reasonable request.

## Abbreviations

NHC: National Health Commission of the People’s Republic of China
lncRNAs: Long non-coding RNAs
ccRCC: Clear cell renal cell carcinoma
IGF2BP3: Insulin-like growth factor 2 mRNA binding proteins
AGAP2-AS1: AGAP2 antisense RNA 1
miR: MicroRNAs
miR-9-5p: 5 Prime of miR family member miR-9
THBS2: Thrombospondin 2
m6A: N6-methyladenosine
PI3K/Akt: Phosphatidylinositol-3 kinase/protein kinase B
TNM: Tumor-node-metastasis
CCK-8: Cell Counting Kit-8
FISH: Fluorescence in situ hybridization
RIP: RNA immunoprecipitation
sh-: Short hairpin RNA-
NC: Negative control
oe-: Overexpression
PCR: Polymerase chain reaction
