# Supplementary figures and images for: LncRNA AGAP2 antisense RNA 1 stabilized by insulin-like growth factor 2 mRNA binding protein 3 promotes macrophage M2 polarization in clear cell renal cell carcinoma through regulation of the microRNA-9-5p/THBS2/PI3K-Akt pathway
Source: Cancer Cell Int. 2023 Dec 18;23:330. doi: 10.1186/s12935-023-03173-5 (PMC10729468; doi:10.1186/s12935-023-03173-5)

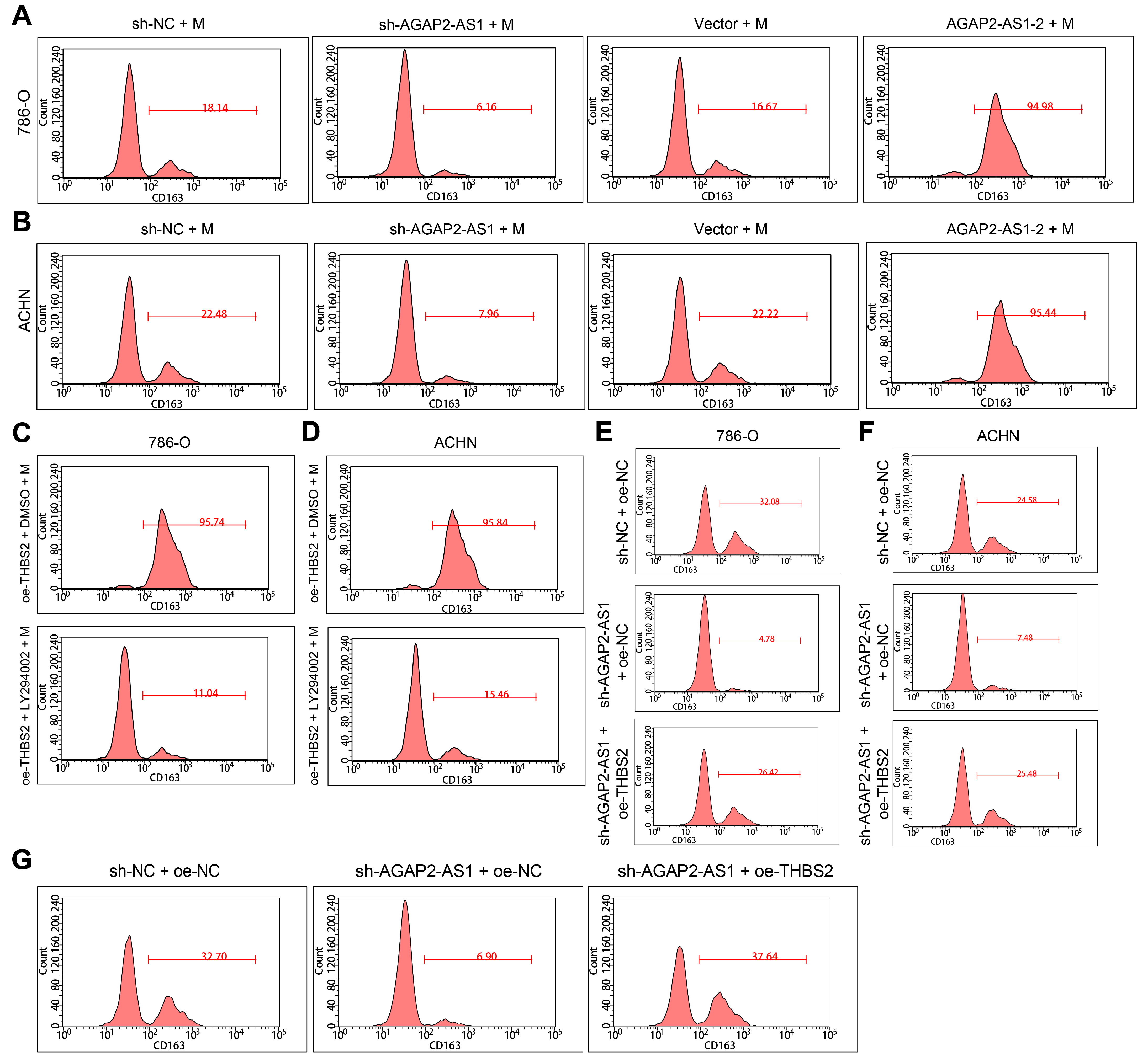

Supplement: Supplementary file 1 — Additional file 1: Figure S1. A and B: Flow cytometry results of macrophage M2 polarization marker CD163 expression following silencing or overexpression of AGAP2-AS1. C and D: Expression of M2 polarization marker CD163 in macrophages following flow cytometry treatment of oe-THBS2, DMSO, and LY294002. E and F: Expression of M2 polarization marker CD163 in macrophages co-cultured with sh-AGAP2-AS1-/oe-THBS2-treated cells by flow cytometry. G: Expression of M2 polarization marker CD163 in macrophages following sh-AGAP2-AS1 and oe-THBS2 treatment analyzed by flow cytometry. [file 12935_2023_3173_MOESM1_ESM.jpg]

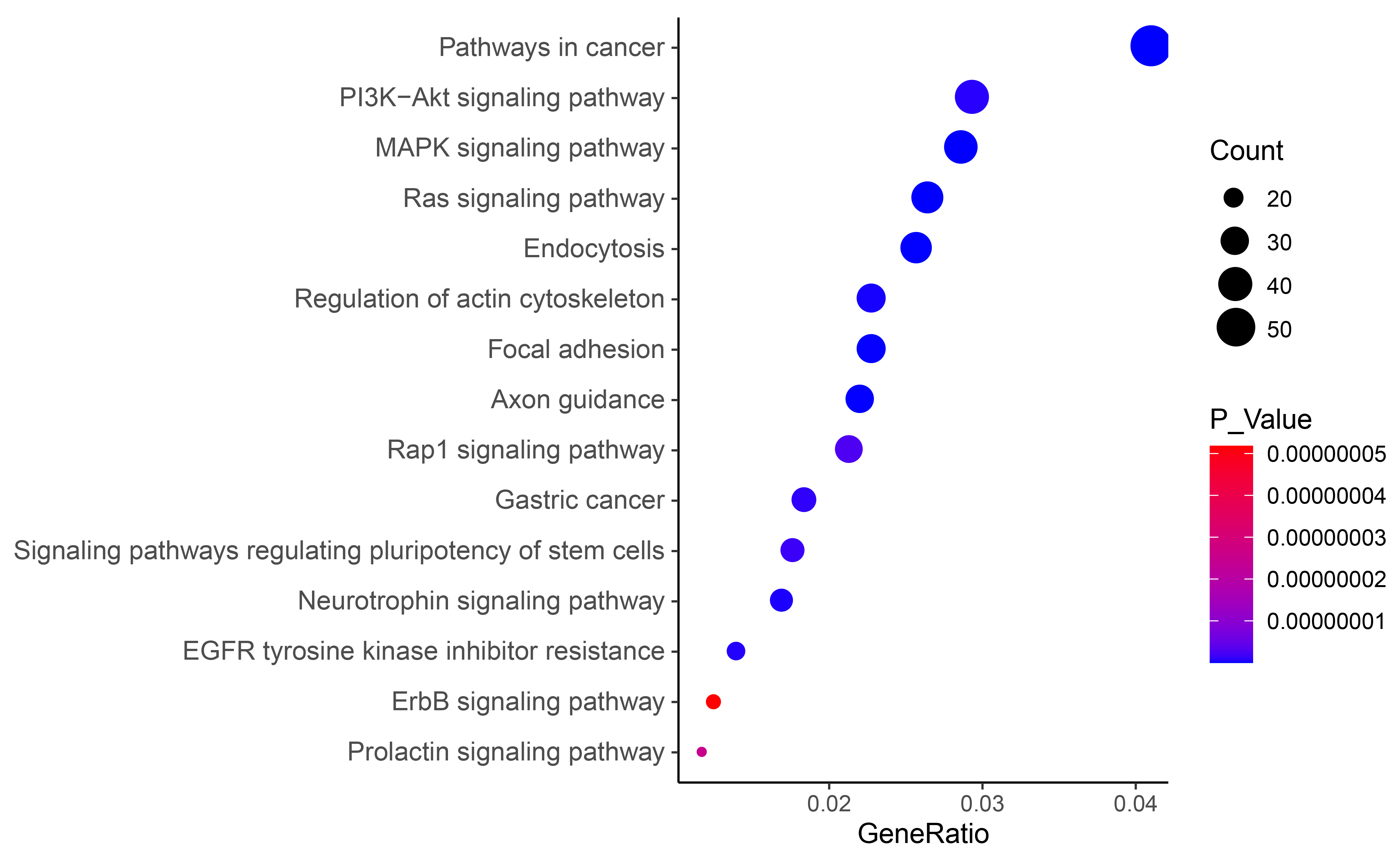

Supplement: Supplementary file 2 — Additional file 2: Figure S2. KEGG pathway enrichment analysis of miR-9-5p target genes. The top 15 results of the KEGG pathway enrichment analysis of miR-9-5p target genes are listed. The y-axis indicates pathways, and the X-axis indicates the ratio of genes enriched in the pathway [file 12935_2023_3173_MOESM2_ESM.jpg]
