# Supplementary material for: LncRNA AGAP2 antisense RNA 1 stabilized by insulin-like growth factor 2 mRNA binding protein 3 promotes macrophage M2 polarization in clear cell renal cell carcinoma through regulation of the microRNA-9-5p/THBS2/PI3K-Akt pathway
Source: Cancer Cell Int. 2023 Dec 18;23:330. doi: 10.1186/s12935-023-03173-5 (PMC10729468; doi:10.1186/s12935-023-03173-5)
Supplement: Supplementary file 3 — Additional file 3: Table S1. Clinical and pathological features of patients (N = 50). Table S2. RT-qPCR primer sequences. [file 12935_2023_3173_MOESM3_ESM.docx]

**Table S1 Clinical and pathological features of patients (N=50)**

| Characteristics | N | AGAP2-AS1 expression | | *P* value |
| --- | --- | --- | --- | --- |
|  |  | High (n=25) | Low (n=25) |  |
| **Age (years)** |  |  |  | 0.5287 |
| <55 | 14 | 8 | 6 |  |
| ≥55 | 36 | 17 | 19 |  |
| **Gender** |  |  |  | 0.1452 |
| Male | 31 | 18 | 13 |  |
| Female | 19 | 7 | 12 |  |
| **Tumor Size (cm^3^)** |  |  |  | 0.0206 |
| <4 | 8 | 1 | 7 |  |
| ≥4 | 42 | 24 | 18 |  |
| **Metastasis** |  |  |  | 0.0007 |
| No | 35 | 12 | 23 |  |
| Yes | 15 | 13 | 2 |  |
| **TNM stage** |  |  |  | <0.0001 |
| I+II | 26 | 4 | 22 |  |
| III | 24 | 21 | 3 |  |

**Table S2 RT-qPCR primer sequences**

| Primers | Sequences (5'-3') |
| --- | --- |
| GAPDH  AGAP2-AS1  miR-9-5p  IGF2BP3  iNOS  TNF-α  Arg-1  IL-10  U6 | Forward: CCACATCGCTCAGACACCAT  Reverse: CCAGGCGCCCAATACG  Forward: TACCTTGACCTTGCTGCTCTC  Reverse: TGTCCCTTAATGACCCCATCC  Forward: TCTTTGGTTATCTAGCTGTATGA  Reverse: Universal reverse primer  Forward: AGTTGTTGTCCCTCGTGACC  Reverse: GTCCACTTTGCAGAGCCTTC  Forward: GCAGAATGTGACCATCATGG  Reverse: ACAACCTTGGTGTTGAAGGC  Forward: GTTCCTCAGCCTCTTCTCCTTC  Reverse: GCTTGTCACTCGGGGTTCGAGA  Forward: TATATCTGCCAAGGATATTGTG  Reverse: AACATCAAAACTTAGATGAATT  Forward: TCTCCGAGATGCCTTCAGC  Reverse: TCAGACAAGGCTTGGCAAC  Forward: AAAGCAAATCATCGGACGAC  Reverse: Universal reverse primer |

Notes: RT-qPCR, reverse transcription quantitative polymerase chain reaction; GAPDH, glyceraldehyde-3-phosphate dehydrogenase; AGAP2-AS1: AGAP2 antisense RNA 1; miR-9-5p, microRNA-9-5p; IGF2BP3, Insulin-like growth factor 2 mRNA binding proteins; iNOS, inducible nitric oxide synthase; TNF-α, tumor necrosis factor-α; Arg-1, Arginase-1; IL-10, interleukin 10.
